# Role of Epicardial Adipose Tissue Secretome on Cardiovascular Diseases

**DOI:** 10.3390/biomedicines11061653

**Published:** 2023-06-07

**Authors:** Sara Leo, Elena Tremoli, Letizia Ferroni, Barbara Zavan

**Affiliations:** 1 Maria Cecilia Hospital, GVM Care & Research, Cotignola, 48033 Ravenna, Italy; sleo@gvmnet.it (S.L.); etremoli@gvmnet.it (E.T.); 2 Department of Translational Medicine, University of Ferrara, 44121 Ferrara, Italy

**Keywords:** epicardial adipose tissue, adipose tissue, inflammation, cardiovascular diseases, extracellular vesicles

## Abstract

Obesity and insulin resistance are associated with the inflamed and defective adipose tissue (AT) phenotype, and are established risk factors for cardiovascular diseases (CVDs). Extracellular vesicles (EVs) are a heterogeneous group of cell-derived lipid membrane vesicles involved in the onset and development of many pathologies, including insulin resistance, diabetes, and CVDs. The inflammation associated with overweight and obesity triggers the transition of the AT secretome from healthy to pathological, with a consequent increased expression of pro-inflammatory mediators. Epicardial adipose tissue (EAT) is a specialized fat depot that surrounds the heart, in direct contact with the myocardium. Recently, the role of EAT in regulating the physiopathology of many heart diseases has been increasingly explored. In particular, the EAT phenotype and derived EVs have been associated with the onset and exacerbation of CVDs. In this review, we will focus on the role of the AT secretome in the case of CVDs, and will discuss the beneficial effects of EVs released by AT as promising therapeutic candidates.

## 1. Introduction

For decades, adipose tissue (AT) was not deeply researched, as it was thought to be merely an energy storage depot. The secretory function of AT was only recognized in the mid-1980s, and an increasing number of scientific works involving adipocytes and AT have been published since then. Currently, AT is accepted as the largest endocrine organ, secreting over 600 proteins [1,2]. To date, numerous adipocyte-derived secreted hormones (leptin, adiponectin, and resistin) and effectors have been demonstrated to modulate a plethora of physiological and pathological metabolic processes, spanning from food intake, gluconeogenesis, glucose uptake, fatty-acid oxidation and insulin resistance, to reproductive axes and immune responses.

CVDs are a group of disorders altering heart functionality, which are characterized by a high fatality rate, and are the driving cause of premature death in humans. Among the main risk factors leading to the onset of CVDs are obesity and diabetes [3,4,5]. These pathologies are associated with a low-grade inflammatory state that differentially affects the homeostasis and the secretome of specific AT depots. This leads to an alteration in the release of extracellular vesicles (EVs) in terms of both number and cargo, which is strictly dependent on the type and state of the cellular and tissue origin [6,7]. The role of epicardial adipose tissue (EAT) in this framework is of particular interest, as it has a significantly different transcriptome and secretome compared to other fat depots, and is in direct contact with the heart muscle [8].

In this review, we will discuss the connection between a dysfunctional AT, and CVDs. Particularly, we will focus on EVs secreted by AT, focusing on the EAT, and we will examine the prospective role of exosomes, the smallest subtype of EVs, as therapeutic candidates in CVDs.

## 2. Adipose Tissue, an Overview

Historically, AT has been divided into two different types: white adipose tissue (WAT), which represents the majority of adipose mass, and brown adipose tissue (BAT), which is unique to mammals, and in humans is located in narrow depots in the upper part of the body, particularly in cervical, axillary, and paraspinal regions [9,10,11]. In WAT depots, during postprandial times, free fatty acids (FFAs) are converted to triacylglycerols (TAGs) and stored as energy to be released after hydrolyzation during an energy deficit. However, in BAT, energy is dissipated through the hydrolysis of TAGs to release glycerol into the bloodstream, while FFAs are partially released, and partially re-esterified. This process, termed “adaptive (non–shivering) thermogenesis”, aims to generate heat and maintain body temperature. Among many different cell types found in the WAT (e.g., mesenchymal stem cells, vascular cells, inflammatory cells, and endothelial cells), the predominant cell type is the adipocyte, which is characterized by a spherical shape with a cytoplasmic large single lipid droplet, and by a very low number of mitochondria. Unlike white ones, brown adipocytes have an ellipsoidal shape, with multiple small lipid droplets, and a much higher number of mitochondria accommodating the uncoupling protein 1 (UCP1), which transfers protons from the mitochondrial intermembrane space into the matrix, uncoupling the electron-transport chain from ATP synthesis, and generating heat [12]. Recently, a new type of fat cell, the beige or brite adipocyte, has been described. These cells rise sparingly within WAT, in response to different stimuli, such as cold exposure and adrenergic stimulation, in a so-called WAT “browning” process [13,14]. Beige adipocytes share some similarities with brown adipocytes, including multilocular lipid cytoplasmic droplets, and a high density of mitochondria expressing UCP1, which enables the thermogenesis function.

WAT is the most representative AT in humans, and can be divided, on the basis of its localization and functions, into subcutaneous adipose tissue (SAT), lying under the reticular dermis and accounting for up to 90% of total mass; and visceral adipose tissue (VAT), which surrounds internal organs and amounts to 10% of the entire AT mass [15,16]. Specialized fat depots can be intimately associated with internal organs, and are able to modify their microenvironment, mainly acting locally. Among other factors, EAT has recently emerged as playing a pivotal role in the onset of CVDs [17,18].

## 3. Epicardial Adipose Tissue

**Figure 1:**
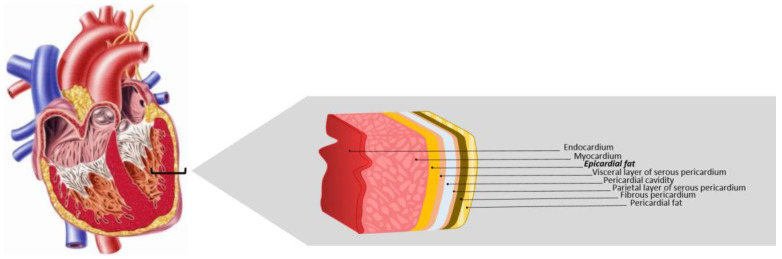
Epicardial adipose tissue. EAT is located between the myocardium and the visceral layer of serous pericardium, directly opposing the cardiac muscle. Adapted from Krishnan [21].

EAT is found between the visceral pericardium and the myocardium, with which it shares a bloodstream, EAT being circulation-dependent on the branches of the coronary arteries [19,20] (Figure 1). The localization of EAT on the heart is distributed between the right ventricle, the anterior wall of the left ventricle, the atrioventricular groove, and the great coronary vessels, reaching the main thickness at the anterior and lateral walls of the right atrium. In physiological conditions, the EAT abundance depends on genetic, epigenetic, and environmental factors, such as pollution, aging, microbiota, and excessive caloric intake. The EAT mass can comprise up to 80% of the heart surface, contributing 20% to its whole mass [19].

### 3.1. Epicardial Adipose Tissue Cellular Components

The cellular components of EAT include copies of white adipocytes, which are smaller than those of VAT, and are specialized in energy storage [22], stroma-vascular cells, neurons, and immune cells including lymphocytes (CD3+), macrophages (CD68+), and mast cells [19]. As an adipose depot, EAT is characterized by a secretory activity that, in physiological conditions, ensures myocardium health [23,24].

The direct interaction of EAT with the myocardium, due to the total absence of fascia between the two tissues and their shared microcirculation, favors the vasocrine and paracrine secretions, with these being of particular importance in explaining the role that EAT plays in the development of cardiovascular pathophysiology. Several studies have demonstrated that a dysfunctional EAT mass expansion, together with its pro-inflammatory state and secretome, is associated with coronary artery diseases (CAD) [25], metabolic syndrome [26], chronic heart failure [27,28], and atrial fibrillation (AF) [29,30,31].

### 3.2. Epicardial Adipose Tissue Functions

EAT performs many different functions. Due to its elasticity and compressibility, it confers onto the coronary artery a mechanical protection against excessive distortion and compression induced by myocardial contractions [20]. Thanks to the similarity to BAT and to the molecular features of beige adipocytes, EAT can defend the myocardium against hypothermia and unfavorable hemodynamic conditions, contributing to cardiac cryoprotection [32,33,34]. Moreover, EAT serves as an energy reservoir for the myocardium, having a high capability for mobilization, deposition, and synthesis of FFAs [35]. It performs a pivotal function in lipid and glucose homeostasis regulation [18], and pro-inflammatory and anti-inflammatory cytokine production, showing a unique expression profile for genes linked to coagulation, endothelial function, apoptosis, immune response, and a specific secretome [18,36,37,38].

## 4. Extracellular Vesicles

EVs are membrane-packed vesicles that can be secreted by many mammalian cell types, and can be found in almost all body fluids, including plasma, saliva, and even breast milk. EVs can be divided into two large subgroups, depending on their dimensions and specific membrane markers. The microvesicles (or ectosomes), ranging from 100 nm to 1000 nm, are positive for CD40 ligand, and Annexin A1 or Annexin V [39,40,41]. The smallest EVs, exosomes, ranging from 40 nm to 100 nm, show increased levels of CD63, CD9, and CD81, among others, along with proteins involved in their biogenesis (e.g., Alix, TSG101, FLOT-1) [42]. The biogenesis of these two classes of vesicles is profoundly different, because larger vesicles are generated by the external budding of the plasma membrane, while exosomes are generated in a multistep process, by inward budding of the endocytic cisternae membrane. Firstly, exosome precursors are accumulated in the cytoplasm into multivesicular bodies (MVBs) that, upon proper stimulation, fuse with the plasma membrane, and release mature exosomes in the extracellular space. The cargo of EVs is enriched in proteins, lipids, and various RNAs (mRNA, miRNA, and circular RNA) that are peculiar for parental cells, and for the developmental and functional state of the generating cells. Once in the extracellular fluids, vesicles are internalized into neighboring or even distant target cells by different mechanisms, and upon content release into the intracellular space, their regulatory effects are exerted. However, it is worth considering that, although many studies are aimed at the identification of the exact content of EVs [43,44], a complete understanding of the intricate biological effects and functionality transferred to recipient cells by the EV cargo has still not been reached. An aberrant production and/or cargo of EVs has been established in the context of many different pathological scenarios, including cancer, diabetes, insulin resistance, and CVDs [45,46,47].

Thus, a multitude of different vesicles are present in extracellular space and in body fluids; however, to date, although several separation methods based on an EV’s specific features have been developed, only a mixed EV population can be isolated.

## 5. Adipose Tissue EVs in Cardiovascular Diseases

Obesity is significantly linked to an increased risk of CVDs, as it is associated with an inflamed and dysfunctional AT phenotype [48,49] that shares some aspects with the classical inflammatory state, such as tissue infiltration by immune cells, and overproduction of inflammatory effectors (e.g., IL-6, IL-1, and TNF-α) [50,51,52], but differs in intensity (being reported as low-grade or subacute) and chronicity [53,54]. The main trait of obese AT inflammation is the increase in macrophage density, and their phenotypic switch from anti-inflammatory M2 to pro-inflammatory M1 cells [55,56,57].

Recent evidence has demonstrated that this inflammatory state is fundamental in the earliest physiological phases of AT adaptation to prolonged, excessive caloric intake. Indeed, infiltrated macrophages not only are responsible for the secretion of the extracellular matrix protein fundamental for the expansion of the AT [58,59], but also play a primary role in the induction of angiogenesis, as demonstrated in mouse models [60]. Moreover, macrophages are able to store TAGs released from dead white adipocytes [61,62], and can modulate lipid trafficking, increasing the lipid buffering capability of adipocytes [63]. However, a further prolongation of the hypercaloric state results in an exceeding capability of the WAT for fat storage, and a gradual alteration in the AT [64], leading to fibrosis, a massive influx of immune cells, an increased production of pro-inflammatory cytokines and chemokines [65,66,67], and a progressive decrease in insulin sensitivity and glucose tolerance in adipocytes [68,69].

The massive increase and remodeling of AT in low-grade inflammatory conditions induced by overweight and obesity is strictly related to the switching of the AT secretome from healthy to unhealthy, with an increase in cytokines and pro-inflammatory-mediator expression. The dysfunctional AT secretome is primarily linked to comorbidities, including hypertension and insulin resistance among others, which in turn lead to the development of CVDs that represent the major cause of death in diabetic people.

The active secretory function of AT has been recognized for some decades, and according to substantial evidence supporting this feature, AT has subsequently been considered the major contributor to whole-body metabolism regulation.

EVs secreted from the cells lying in the AT (e.g., immune cells, mesenchymal stem cells, and endothelial cells) can exert their functions both through the endocrine and the paracrine ways, acting also on distal organs such as the skeletal muscle, liver, brain, pancreas, and heart. High throughput sequencing and proteomic analysis of AT-derived EVs identified a plethora of miRNAs and proteins involved in the modulation of many different cell processes [70,71].

EVs released by the AT of ob/ob mice were found to induce insulin resistance and macrophage activation in a TLR-4-dependent manner, by increasing the migration and the protein levels of the pro-inflammatory cytokines IL-6 and TNF-α [72]. The pro-inflammatory feature of dysfunctional EVs isolated from human adipose mesenchymal stem cells (ADMSCs) was also demonstrated in a recent paper by Eirin et al. [73]. The authors stated that ADMSCs-EVs of obese individuals are enriched in miRNA-regulating, pro-inflammatory, and apoptosis-signaling cell proliferations, at the expense of a reduction in miRNA-cargo-regulating cell proliferations, and angiogenic pathways (the gene involved in angiopoietin signaling, VEGF and its receptor KDR). Differences in ADMSCs-EV cargo between lean and obese individuals are supposed to be the reason for the impaired resilience of renal tubular cells to ischemic injury. The limited pro-angiogenic potential of ADMSCs isolated from obese subjects was already investigated in a previous study carried out on obese and non-obese participants [74]. In particular, ADMSCs-EVs isolated from both VAT and SAT, although comparable in number and size, have limited VEGF and MMP-2 metalloproteinase protein levels and miRNA-126 expression. Furthermore, EVs isolated form the VAT of high-fat-diet (HFD) -induced obese mice were demonstrated to play a pivotal pro-atherosclerotic role in the regulation of macrophage foam cells and M1 polarization by NK-kB activity modulation [75]. Dysfunctional miRNA-130b-3p-enriched EVs from diabetic patients were also proven to exacerbate the ischemic/reperfusion injury in vivo and in vitro, through the negative regulation of AMPK suppressing multiple anti-apoptotic and cardio-protective molecules in cardiomyocytes [76].

## 6. Epicardial Adipose Tissue EVs in Cardiovascular Diseases

The inflammatory conditions that characterize different pathologies such as obesity and diabetes are responsible for the fibrotic and inflammatory state of the heart [77,78]. Recently, the epicardial fatty depot has been considered as an important regulator of cardiovascular health status, and its pathological phenotype has been associated with the onset and exacerbation of CVDs [8,31]. As evidence of this, the worsening of coronary atherosclerosis disease (CAD) has been shown to be significantly associated with a reduced adiponectin mRNA level, and with an increased IL-6 mRNA level in EAT [79]. EAT thickness has been correlated with insulin resistance and many other risk factors of cardiovascular pathologies [80,81,82], but the exact mechanism linking EAT to cardiac dysfunction has yet to be fully elucidated. Lately, the role of the EAT secretome, particularly of EVs released by the depot, in the pathogenesis of CVD, has been greatly explored. From the latest studies published in the literature, the hypothesis has been established of a unique secretome characterizing pathological EAT that is involved in the development and propagation of CVD. 

Exosomal miRNA-802-5p released by hypertrophic 3T3-L1 cells has been demonstrated to cause insulin resistance in neonatal rat ventricular myocytes through the reduction in the expression of intracellular HSP60, a mitochondrial chaperone already known to be involved in CVDs among obese and diabetic patients [83]. Insulin resistance has been considered the main factor linking the diabetic conditions to the occurrence of many CVDs and heart failure. The close anatomic proximity between the EAT and the myocardium could be the basis of the paracrine crosstalk between the two tissues, explaining a possible mechanism through which EAT impairs insulin signaling, and consequently induces the structural and functional alteration in cardiomyocytes. Moreover, in CAD patients, the EAT microenvironment plays a pivotal role in modulating the cargo of EAT exosomes that in turn is responsible for an impaired adipogenic differentiation in stem cells lying in the depot [84]. Indeed, although epicardial adipose stem cells (EASCs) from CAD and non-CAD patients have an identical adipogenic potential, Wankei Y. et al. have recently demonstrated that it decreases significantly after exposure to EAT-derived exosomes of CAD subjects. This evidence indicates that various factors triggering CAD (e.g., insulin resistance and inflammation) are responsible for an alteration in the EAT secretome. In particular, the effects observed were ascribed to the down-regulation of Neuronatin protein targeted by miR-3064-5p, enriched in EAT-exosomes of CAD patients.

In addition, a direct role of EVs released by EAT in AF onset and propagation was demonstrated for the first time, in a very comprehensive study by Shaihov-Teper et al. [85]. AF is a multifactorial atrial arrhythmia, very often interconnected with CVD. Histological examinations of EAT explant from AF and non-AF patients were analyzed, and an excess of extracellular matrix deposition, and inflammatory cell infiltrations, were found in AF patients. The EVs isolated from EAT explant cultured in vitro have revealed that the vesicles from AF (AF-EVs) were enriched in pro-inflammatory cytokines (IL-6, IL-1a, TNF-α, IL-4), with lower levels of IL-10 (anti-inflammatory and pro-fibrotic cytokine), VEGF and soluble VEGF receptor. The proteomic profile of AF-EVs corroborated the pro-inflammatory and pro-fibrotic outline that may be ascribed to the upregulation of miR-146b, and to a reduction in miR-133a and miRNA-29a expression. Furthermore, the pro-arrhythmic feature of AF-EVs in a two-dimensional hiPSC-derived cardiac cell sheet was demonstrated. Remarkably, the AF-EV profile was independent from the method used for isolation; both ultracentrifugation and size exclusion chromatography isolation preserve the EV signature. Other possible EAT-EVs-dependent pathways implicated in AF pathogenesis involve circular RNAs. Circular RNAs are non-coding RNAs characterized by a closed-loop structure that provides a high stability released in the extracellular space within exosomes [86,87]. They regulate gene expression, acting as sponges by buffering specific miRNA, and impeding their target gene’s silencing [86,88,89]. Recently, the circular RNAs from the EAT of patients with AF were profiled, and an unique expression profile was shown [90]. The reconstruction of a circular RNA–miRNA–mRNA interactional network has demonstrated that hsa_circRNA_000932 and hsa_circRNA_0078619 modulate the expression of many genes involved in the CVD frame, through the interaction with various miRNAs such as miR-103a-2-5p and miR-199a-5p, providing a direct link between EAT exosomal circular RNA, and the structural and functional remodeling of the heart in AF development.

The contribution of the secretome deriving from EAT and AT in inflammatory processes and in the onset of CVDs are summarized in Table 1.

## 7. Functionalized Adipose Tissue EVs in Cardiovascular Diseases

Stem-cell transplantation has been extensively investigated as a promising therapeutic candidate in the treatment of many different diseases, including CVDs [93,94,95,96]. However, results obtained from clinical studies are limited by the poor survival rate of stem cells in the ischemic and inflamed environment, and by their malignant potency [97]. In light of increasing knowledge about the functionality of EVs released from AT, many studies have recently focused on the beneficial effects of EVs derived from stem cells, rather than on stem cells, per se [98,99,100,101,102].

Cui et al. [103] have demonstrated the protective role of exosomes isolated from rat AT mesenchymal stem cells in the myocardial viability of rat H9c2 cardiomyocytes exposed to hypoxia/reoxygenation (H/R). The authors confirmed these results in vivo, proving that the infusion of ADMSCs-EVs in a rat I/R model reduces the myocardial infarction (MI) size, and the serum level of the principal myocardial enzymes induced by I/R damage (creatine kinase–myocardial band, CK–MB, lactate dehydrogenase, LDL, and cardiac troponin I). As demonstrated in vitro on H9c2 cells, the activation of the Wnt/beta-catenin pathway is behind the reduction in cell apoptosis (the principal mechanism of cell death in I/R injury responsible for an altered cardiac function) and the increase in cell viability.

Due to the simplicity of their isolation method, and to the secretion of mediators that favor tissue repair, mesenchymal stem cells (MSCs) have become one of the most promising cell-therapy tools employed in CVDs. MSCs can be isolated not only from AT but also from bone marrow (BMMSCs) and umbilical cord blood (UCMSCs) [104,105]. The cardio-protective effects of both in vitro cultured human MSCs, and exosomes derived from the same maternal cells, were investigated in rat models of MI [106]. The authors demonstrated a reduction in the infarction area and the apoptotic rate of damaged cardiomyocytes, and an increase in the microvascular density after MSC transplantation or MSC-derived exosome injection. Both ADMSCs and ADMSC-derived exosomes (ADMSC-exos) exert the most significant effect. These data enforce the role of ADMSC-exos as promising candidates for myocardial tissue repair after MI-induced damage.

In addition to the anti-apoptotic and pro-angiogenic properties, ADMSC-exos also display the ability to promote the polarization of macrophages towards the M2-anti-inflammatory phenotype, targeting the S1P/SK1/S1PR1 pathway, and leading to a down regulation of the MI-induced inflammatory factor expression, and to a concomitant improved cardiac function [107].

The coronary endothelium plays an important role in the repair response to ischemic injury, as myocardial reperfusion and the de novo synthesis of new blood vessels are fundamental in the recovery process. Carter et al. [108] have demonstrated that EVs produced by adipose microvasculature or human coronary artery endothelial cells (HCAEC) exposed to pro-inflammatory conditions resembling the AT of obese humans, ischemic injuries, or CVDs, trigger a defective EV-dependent reduction in barrier function and proliferation in naive HCAEC, which could be partially attributed to a dysfunctional miRNA EV cargo. Conversely, EV isolated from HCAEC in physiological conditions ameliorate the cell response to wounding, and the permeability of HCAEC monolayers.

The beneficial effects of exosomes isolated from AT on myocardial repair after hypoxic and ischemic injury can be improved, functionalizing exosomes with factors that are known to participate in the myocardial function restoration. It was the case that exosomes isolated from miR-126 overexpressed ADMSCs (miR-126 ADMSCs-exos) [109]. The effects of miR-126 enriched ADMSCs-exos were investigated both in vitro and in vivo on acute MI rat models. The in vitro results on H9c2 myocardial cells shown a reduction in the expression levels of inflammatory factors (IL-1b, IL-6, and TNF-α), a decrease in the expression of fibrosis-related proteins, and an increase in cell viability under ischemic conditions. Furthermore, miR-126 ADSCs-exos promote angiogenesis and migration of peripheral endothelial progenitor cells (EPCs) under hypoxic conditions. These potentially therapeutic effects on heart function after ischemic injury were also confirmed in acute MI rat models. Similarly, exosomes from ADMSCs overexpressing SIRT1 (deacetylase involved in myocardial injury repair during diabetes [110]) have a protective effect on acute MI. EPCs of acute MI treated with SIRT1-ADMSCs-exos showed an increased expression of CXCL12 and Nrf2 that promoted migration and tube formation. Moreover, the injection of SIRT1-ADMSCs-exos decreased infarct size and myocardial inflammation, and promoted angiogenesis in the mouse model [111].

## 8. Conclusions

Since the recognition of the secretory function of AT, an increasing number of scientific works involving adipocytes and AT have been published. Obesity is associated with chronic low-grade AT inflammation, which impacts both the number and cargo of EVs released from adipose depots and, in turn, impairs the physiological regulation of many processes with which they are involved. A growing body of evidence from the literature demonstrates a direct role played by AT-isolated EVs in the pathophysiology of CVDs. EAT is the VAT of the heart, and is characterized by the direct interaction, and the shared bloodstream, with the myocardium, features that can promote the vasocrine and paracrine secretion. As for AT, the EAT secretome switches from healthy to unhealthy in inflammatory conditions.

Although, as pointed out in recent publications [112,113,114], the number, the size, and the molecular cargo of circulating EVs in healthy conditions are highly variable between individuals, and are strongly influenced by different cellular and extracellular stimuli, complicating the identification of hallmarks of disease, EAT-EVs have attracted great interest in terms of providing a prognostic and diagnostic tool for CVDs.

Lately, the role of ADSCs-EVs not only as simply biomarkers, but also as a potent therapeutic tool in CVD treatment, has been investigated in order to overcome the main constraints of cell-based therapy. Indeed, the efficacy of the stem-cell transplantation approach in CVD handling is limited by several factors, such as the lack of blood supply to ischemic injured area, the poor survival rate, and the potential risk of malignant transformation. ADSCs-EVs are easily accessible, and can be functionalized with effectors involved in CVD pathophysiology, in order to ameliorate their therapeutic effect. However, many questions concerning the application of exosomes as an alternative strategy to the cell-based approach are still open, and remain to be solved. First of all, the impossibility of obtaining a relative pure EV population. Indeed, to date, several techniques aimed at the isolation of a pure EV population based on EV characteristics have been developed, but none has been successful. Secondly, the short stability of EVs has limited the research to short-term studies, meaning that the long-term effects remain unexplored. A final limitation is the dependence of the EV cargo on the donor cell conditions, and the potentially reduced therapeutic efficacy due to the competition between the uptake of healthy and unhealthy EVs. Despite the limitations mentioned above, ADSCs-EV will for sure remain the focus of new diagnostic and therapeutic applications in CVDs.

## Figures and Tables

**Table 1:** The adipose tissue (AT) and epicardial adipose tissue (EAT) secretomes contribute to inflammation and CVD occurrence.

Tissue	Effects	Mediators	Ref
AT	insulin resistance and macrophage activation	IL-6; TNF-α	[72]
	M1 polarization and formation of macrophage foam cells	IL-6; TNF-α	[75]
	Adipogenesis	miR-450a-5p	[70]
	macrophages M2/M1 phenotypic switching and insulin resistance	TNF-α; MCP-1	[55,91,92]
	secretion of extracellular matrix protein	plasminogen activator; proteases; type VI collagen	[58,59]
	angiogenesis	VEGF; MMPs; SDF-1; miR-126	[60,71,73,74]
	cell cycle and apoptosis regulation	hsa-miR-222-5p; miR-888-5p; hsa-miR-6752-5p; has-miR-6338-3p; miR-130b-3p	[73,76]
EAT	insulin resistance	miR-802-5p	[83]
	arterial damage	adiponectin; IL-6	[79]
	adipogenic differentiation	miR-3064-5p	[84]
	inflammation, fibrosis, and apoptosis promotion	IL-1α; IL-6; TNF-α; IL-4; VEGF; IL-10; sFLT-1	[85]
	Modulation of inflammation and cell proliferation	hsa_circ_0099634; hsa_circ_0000932; hsa_circ_0097669; hsa_circ_0135289; hsa_circ_0098155; hsa_circ_0079672	[90]

## Data Availability

Not applicable.
